# Transient Receptor Potential Channels and Botulinum Neurotoxins in Chronic Pain

**DOI:** 10.3389/fnmol.2021.772719

**Published:** 2021-10-29

**Authors:** Eun Jin Go, Jeongkyu Ji, Yong Ho Kim, Temugin Berta, Chul-Kyu Park

**Affiliations:** ^1^Department of Physiology, Gachon Pain Center, Gachon University College of Medicine, Incheon, South Korea; ^2^Gachon University College of Medicine, Incheon, South Korea; ^3^Department of Anesthesiology, Pain Research Center, University of Cincinnati Medical Center, Cincinnati, OH, United States

**Keywords:** chronic pain, migraine, TRPV1, TRPA1, exocytosis, botulinum neurotoxin

## Abstract

Pain afflicts more than 1.5 billion people worldwide, with hundreds of millions suffering from unrelieved chronic pain. Despite widespread recognition of the importance of developing better interventions for the relief of chronic pain, little is known about the mechanisms underlying this condition. However, transient receptor potential (TRP) ion channels in nociceptors have been shown to be essential players in the generation and progression of pain and have attracted the attention of several pharmaceutical companies as therapeutic targets. Unfortunately, TRP channel inhibitors have failed in clinical trials, at least in part due to their thermoregulatory function. Botulinum neurotoxins (BoNTs) have emerged as novel and safe pain therapeutics because of their regulation of exocytosis and pro-nociceptive neurotransmitters. However, it is becoming evident that BoNTs also regulate the expression and function of TRP channels, which may explain their analgesic effects. Here, we summarize the roles of TRP channels in pain, with a particular focus on TRPV1 and TRPA1, their regulation by BoNTs, and briefly discuss the use of BoNTs for the treatment of chronic pain.

## Introduction

Pain is defined as an unpleasant sensory and emotional experience associated with or resembling that associated with actual or potential tissue damage (Kuner, 2010). Acute pain is transient and beneficial and mainly functions as a protective warning for the body. In contrast, chronic pain is a persistent and debilitating condition for which there are few treatment options (Treede et al., 2015). Chronic pain manifests in symptoms such as spontaneous pain, hyperalgesia (i.e., increased pain from a stimulus that normally provokes pain), and allodynia (i.e., pain due to a stimulus that does not normally provoke pain) (Treede et al., 1992). Chronic pain has numerous etiologies, including arthritis-induced inflammatory pain, cancer pain, diabetic neuropathy, spinal cord injury, and nerve injury. Although pain results from the complex processing of neural signals at different levels of the nervous system (Costigan et al., 2009; Reichling and Levine, 2009), targeting the beginning of the pain pathway and aim potential treatments directly at receptors and ion channels expressed in nociceptors (i.e., peripheral sensory neurons that detect pain) seems to be a logical strategy for developing novel analgesics.

Among all the receptors and ion channels expressed in nociceptors, transient receptor potential (TRP) ion channels have been extensively studied for their participation in various acute and chronic pain conditions (Lumpkin and Caterina, 2007), with TRPV1 and TRPA1 having emerged as especially promising targets for analgesics. Both TRPV1 and TRPA1 act as polymodal detectors in nociceptors, and their activation by endogenous mediators and natural products (e.g., capsaicin and mustard oil, respectively) can elicit pain (Scholz and Woolf, 2002; Jardin et al., 2017). Further proof of the involvement of these ion channels in pain is provided by their well-reported transcriptional, translational, and trafficking regulation, leading to nociceptor hyperexcitability and pain after inflammation or nerve injury (Hucho and Levine, 2007). For these reasons, several pharmaceutical companies have been conducting clinical trials of TRPV1 and TRPA1 antagonists. However, these antagonists, mainly those of TRPV1, have demonstrated undesirable adverse side effects such as hyperthermia (Gavva et al., 2008), reduced heat pain threshold, and reduced taste perception (Patapoutian et al., 2009). These findings suggest that additional strategies should be pursued to target TRP channels and alleviate pain.

Botulinum neurotoxins (BoNTs) have demonstrated analgesic effects in various animal models of acute and chronic pain, and the use of BoNT serotype-A (BoNT/A) is currently approved for the treatment of chronic migraines (Matak et al., 2019). However, knowledge of the mechanisms by which BoNTs inhibit pain is currently limited. Although it has been suggested that such inhibition is driven by the modulation of pro-nociceptive neuropeptides, BoNTs have been shown to interact with and regulate TRP channels, which may underlie their analgesic effects.

In this succinct review, we focus on TRPV1 and TRPA1 as essential nociceptive mediators, present the underlying mechanisms of their interaction and regulation by BoNTs, and finally propose BoNTs as a novel strategy to treat various acute and chronic pain conditions.

## Transient Receptor Potential Channels in Chronic Pain

TRP channels are non-selective ion channels mostly located on the plasma membrane of various cell types and are divided into six main groups: TRPV (vanilloid), TRPA (ankyrin), TRPM (melastatin), TRPC (canonical), TRPP (polycystin), and TRPML (mucolipin) (Chung and Caterina, 2007). The first suggestion that TRP channels were key receptors involved in sensory transduction emerged from the identification of TRPV1 as a capsaicin- and heat-activated ion channel (Rosenbaum and Simon, 2007). Cutaneous injection of capsaicin, the active ingredient of chili peppers, induces pain-like sensations such as burning, itching, piercing, pricking, and stinging (O’Neill et al., 2012). Similarly, several pungent chemicals (e.g., mustard and cinnamon, but not capsaicin) activate TRPA1, which can also lead to pain-like sensations (Patapoutian et al., 2009). TRP channels can participate in acute and chronic pain through transcriptional and translational regulation, post-translational changes, and altered trafficking (Patapoutian et al., 2009). Among neurons, nociceptors uniquely express TRPV1 and TRPA1, which make them particularly interesting in the chronic pain states, since their expression and function after inflammation and nerve injury contribute to the pathological pain states by increasing sensitivity to nociceptive stimuli (i.e., peripheral sensitization) (Hucho and Levine, 2007).

During inflammation, both TRPV1 and TRPA1 transcripts are increased through the neuronal C-C chemokine receptor type 2 (CCR2), which is activated by the release, by macrophages, of pro-inflammatory protein-1α (MIP-1α/CCL3) (Jung et al., 2008). Similarly, oxidative stress products generated by tissue damage and inflammation lead to increased expression and function of TRP channels, resulting in neuronal hyperexcitability and pain (Hucho and Levine, 2007). It has also been shown that pro-inflammatory mediators such as tumor necrosis factor (TNF)-α engage both the PKC and PKA signaling pathways, altering the activity and function of TRPV1 (Premkumar and Ahern, 2000; Chuang et al., 2001; Vellani et al., 2001; Bhave et al., 2002, 2003; Crandall et al., 2002; Numazaki et al., 2002, 2003; Prescott and Julius, 2003), whereas nerve growth factor (NGF), acting via p38 mitogen-activated protein kinases (MAPKs), increases the translation of TRPV1 in the cell body, and promotes its trafficking to the peripheral terminals (Ji et al., 2002). In addition to the complexity of TRP channels, their trafficking to the cellular membrane is followed by further activation of second-messenger pathways and post-translational modifications such as channel phosphorylation and glycosylation (Morenilla-Palao et al., 2004; Zhang et al., 2005).

Nerve injury also leads to regulation of TRP channels. Although the expression and function of TRPV1 are altered after spinal nerve ligation, with decreased TRPV1 expression in injured neurons likely due to trophic support (Michael and Priestley, 1999), increases in TRPV1 and TRPA1 expression in neighboring non-injured neurons are accompanied by hyperalgesia and allodynia (Hudson et al., 2001; Obata et al., 2005). It has been suggested that the increase in TRP channel expression in non-injured neurons is driven by the release of neuropeptides, growth factors, and pro-inflammatory mediators from injured neurons (Fukuoka et al., 2001; Sexton et al., 2014). Indeed, TNF-α alone has been shown to increase the fraction of dorsal root ganglion (DRG) neurons expressing TRPV1 (Hensellek et al., 2007). Likewise, a study reported TRPV1 and TRPA1 trafficking in calcitonin gene-related peptide (CGRP)-releasing vesicles, induced by TNF-α via membrane fusion mediated by soluble N-ethylmaleimide-sensitive factor attachment protein receptors (SNAREs) in trigeminal ganglion neurons (Meng et al., 2016).

Although TRP channels convert thermal, chemical, and noxious stimuli into electrical activity on the peripheral terminals of sensory neurons, they are also found on the synapses in the central terminals of nociceptors projecting into the dorsal horn of the spinal cord (Patapoutian et al., 2009). Interestingly, activation of synaptic TRPV1 and TRPA1 by intrathecal injections of capsaicin and mustard oil, respectively, results in an increase in synaptic release of both glutamate and neuropeptides (Raisinghani et al., 2011). Increased expression of TRPV1 in synaptic terminals after nerve injury leads to increased release of inflammatory neuropeptides such as CGRP and substance P (SP), which also enhance glutamatergic neurotransmission and pain (Kanai et al., 2005; Lappin et al., 2006; Lee and Kim, 2007; Spicarova et al., 2011).

These results suggest a possible role of TRP channel inhibitors, similar to that of BoNTs, as synaptic modulators, by which they can reduce depolarization and control calcium influx and synaptic vesicle exocytosis. Unfortunately, direct inhibition of TRP channels, such as TRPV1, is associated with thermoregulation and adverse side effects. However, targeting the synaptic function of TRP channels and exocytosis may offer a novel and safer therapeutic approach to treat acute and chronic pain.

## Botulinum Neurotoxin: Inhibition of Exocytosis

BoNTs are among the most potent biological toxins produced by neurotoxigenic strains of anaerobic and spore-forming bacteria of the genus *Clostridium* (Mohanty et al., 2001). However, local injection of a small amount of BoNTs is safe and has a wide spectrum of applications for both therapeutic and cosmetic indications (Truong et al., 2009; Dressler, 2012). BoNTs have been traditionally classified into seven serotypes distinguishable with animal antisera and designated with the letters A to G, among which BoNT/A is a commercially available human indication (Simpson, 1981). BoNTs are typical AB-structured toxins, consisting of a heavy chain with membrane acceptor–binding and translocation domains, and a smaller light chain with a catalytic domain that mediates the cytosolic proteolytic activity of these neurotoxins.

The basis of BoNT therapeutic indications is the neuronal inhibition of exocytosis, as they are known for their cleavage of synaptic components of the SNARE complex proteins, thus blocking the release of neurotransmitters, such as CGRP, SP, and glutamate (Nakov et al., 1989; McMahon et al., 1992; Pirazzini et al., 2017). The inhibition of exocytosis by botulinum toxin involves three steps: binding, internalization/translocation, and cleavage of the target (Schiavo et al., 2000). Neuronal tropism is due to a high-affinity interaction of the BoNT heavy chain with double acceptors consisting of gangliosides and synaptic vesicle 2 (SV2A-C) protein isoforms expressed on the extracellular side of the neuronal membrane (Muraro et al., 2009). Internalization of BoNTs occurs via endocytosis, and in the endosome the light chain dissociates from the heavy chain by reduction of the disulfide bridge, being then released into the cytoplasm by an energy- and pH-dependent pore-forming process (Pirazzini et al., 2017). In the cytoplasm, the BoNT light chain cleaves one of the proteins that make up the SNARE complex: synaptobrevin or vesicle-associated membrane proteins (VAMPs), syntaxin-1, and synaptosomal-associated protein 25 (SNAP-25) (Sudhof and Rothman, 2009). This cleavage is highly specific to each BoNT serotype and directed toward unique peptide bonds within the sequence of their respective SNARE protein targets (Pirazzini et al., 2017); for instance, it has been reported that BoNT/A cleaves 9 amino acids at the C-terminus of SNAP-25 (Lebeda et al., 2010). This cleavage results in reduced affinity of the intracellular Ca^2+^ sensor synaptotagmin to SNAP-25, thus impairing exocytosis and the synaptic release of neurotransmitters (Tang et al., 2006; Pan et al., 2009), which, in our context, propagate pain.

Although inhibition of exocytosis and prevention of neurotransmitter release are fundamental mechanisms underlying the neuronal effects of BoNTs, new mechanisms are emerging to explain the specific and potent analgesic effects of these toxins, including their interactions with and regulation of TRP channels.

## Botulinum Neurotoxin: Inhibition of TRP Channels

Sensory information is not equally affected by BoNTs, and it has been reported that BoNT/A mostly alleviates pathological inflammatory pain and mechanical sensation (Cui et al., 2004; Bach-Rojecky et al., 2005; Luvisetto et al., 2006, 2007; Park et al., 2006). This suggests distinct neuronal tropisms for specific sensory neurons. It is also possible that BoNT/A may favor particular sensory neurons with high expression of SV2 proteins that facilitate its entry (Yiangou et al., 2011). Yet another interesting hypothesis stems from the observation that BoNT/A interacts with TRPV1 both structurally and functionally (Li and Coffield, 2016), and therefore selectively enters TRPV1-expressing sensory neurons. Indeed, it has been reported that SNAP-25 cleavage and the analgesic effects of BoNT/A were prevented in animals following denervation of TRPV1-expressing sensory neurons achieved with high doses of capsaicin, an agonist of TRPV1 (Apostolidis et al., 2005; Camprubi-Robles et al., 2009; Shimizu et al., 2012). In line with this preclinical observation, neuropathic pain patients with lower thermal deficits, and thus presumably with lower impairment of TRPV1-expressing sensory neurons, responded better to BoNT/A treatment (Attal et al., 2016).

**FIGURE 1:**
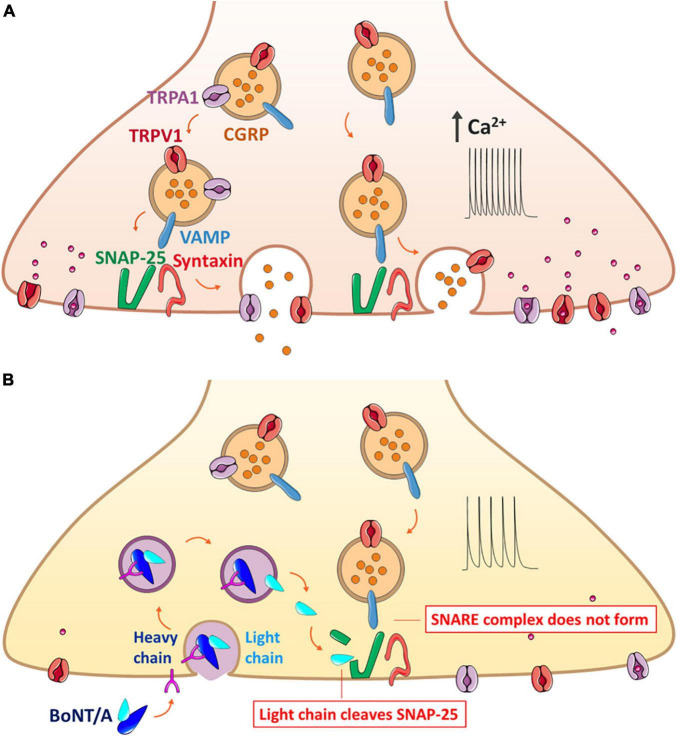
Mechanism of BoNT/A action on the exocytosis of neuropeptide CGRP that forms the SNARE complex along with trafficking of TRPV1 and TRPA1 on the same synaptic vesicle. **(A)** Release of CGRP due to nerve injury and inflammation. The CGRP-containing vesicle, packaged with co-expressed TRPV1 and TRPA1 along with the VAMP protein, moves toward the plasma membrane for the synaptic fusion with SNAP-25 and syntaxin anchored in the plasma membrane. Overexpression of such channels evokes the hyperexcitability of the sensory neurons, ultimately contributing to hyperalgesia and allodynia. **(B)** Blockage of CGRP release by BoNT/A through the prevention of the complete assembly of the synaptic fusion SNARE complex. BoNT/A binds to the cell membrane and enters the sensory neuron by endocytosis; the light chain is translocated to the cytoplasm and claves specific SNAP-25 sites, resulting in inhibition of both the exocytosis of neuropeptides and the surface delivery of TRPV1 and TRPA1. BoNT/A, botulinum neurotoxin serotypes A; CGRP, calcitonin gene-related peptide; DRG, dorsal root ganglion; SANP-25, synaptosomal-associated protein of Mr = 25k; TRP, transient receptor potential; VAMP, vesicle-associated membrane protein or synaptobrevin.

BoNTs reduce the expression of TRP channels in sensory neurons (Figure 1). It has been reported that BoNT/A treatment decreases TRPV1 expression in sensory neurons projecting from the dura mater (Shimizu et al., 2012) and significantly reduces the overexpression of injury-induced TRPV1 protein, but not mRNA, in sensory neurons of the DRG (Xiao et al., 2013). It has been proposed that BoNT/A might block the translocation of TRPV1 to the sensory neurons, as reduced surface expression of the TRPV1 protein was observed *in vivo* and *in vitro* (Gazerani et al., 2006; Tugnoli et al., 2007; Meng et al., 2009; Wang et al., 2011). Another *in vitro* study using a recombinant chimera of BoNT/A and BoNT/E also showed a decrease in TNF-α-dependent TRPV1 and TRPA1 protein translocation to the cellular membrane of sensory neurons (Wang et al., 2017; Nugent et al., 2018).

BoNTs may interfere with TRP channel protein translocation by cleavage of SNAP-25, impairing both synaptic Ca^2+^ concentration and exocytosis (Meng et al., 2009; Meng et al., 2014). Cleavage of SNARE proteins by BoNTs has been demonstrated in sensory neurons. In particular, BoNT/A has been reported to impair exocytosis and recruitment of the TRPV1 receptor to the plasma membrane of nociceptors after stimulation with capsaicin (Gazerani et al., 2006; Gazerani et al., 2009) or mustard oil (Paterson et al., 2014). TRPA1 contributes to mechanical currents in the plasma membrane, and it has been proposed that BoNT/A may also decrease the activity of mechanosensitive receptors and TRPA1 in dural afferents (Burstein et al., 2014; Paterson et al., 2014).

Altogether, these studies demonstrated that BoNTs have distinct tropism toward TRPV1-expressing sensory neurons and can lead to decreased expression and translocation of TRP channels, resulting in a reduction in synaptic Ca^2+^ concentration, release of neurotransmitters, and alleviation of pain. Table 1 shows a list of published studies in which the analgesic effect of BoNT is achieved via modulation of TRP channels in various types of pain.

**TABLE 1:** Analgesic effects of BoNTs through TRP channels in various types of pain.

**References**	**BoNT serotype**	**Administration site/Doses**	**Pain type**	**TRP channel**	**Action duration**	**Results**
Shimizu et al. (2012)	BoNT/A	Subcutaneous (0.25–5 ng/kg) in rat TGN	Nociceptive pain	TRPV1	2–14 days	- SNAP-25 cleavage - ↓ TRPV1 protein expression - ↓ Nociceptive behaviors - Blockage of TRPV1 trafficking
Xiao et al. (2013)	BoNT/A	Intraplantar (10 or 20 U/kg) in rat	Neuropathic pain (L5 VRT)	TRPV1	3–21 days	- Reversed hyperalgesia - ↓ TRPV1 overexpression
Luvisetto et al. (2015)	BoNT/A	Subcutaneous in the inner side of the medial part of hindlimb thigh (15 pg) of mice ** pre-treated	Nociceptive pain	TRPV1 TRPA1	21 days	- ↓ Nociceptive behaviors
Fan et al. (2017)	BoNT/A	Percutaneous in tibial-tarsal hind joint (2.5–25 U/kg) of rats	Adjuvant-arthritis pain	TRPV1 TRPA1	3–14 days	- ↓ mechanical allodynia and thermal hyperalgesia - ↓ Protein levels of TRPV1 in DRG - ↓ TRPV1 expression in DRG - ↓ Percentage of TRPV1-positive neurons with CGRP
Wang et al. (2017) and Nugent et al. (2018)	LC/E-BoNT/A	Intraplantar (25–75 U/Kg) in rat	Neuropathic pain (SNI)		14 days	- Blockage of CAP-evoked CGRP release - Greater analgesic effect than BoNT/A or pregabalin (short-acting pain modulator) - Sustained and prolonged effect by a second injection
				TRPV1 TRPA1		- ↓ Functional activities in TRPV1/A1 with no basal surface contents of rat DRG - ↓ TNF-α-dependent surface trafficking of TRPV1/A1 and calcium influx in rat DRG

CAP, capsaicin; CGRP, calcitonin gene-related peptide; DRG, dorsal root ganglion; SANP-25, synaptosomal-associated protein of Mr = 25k; SNI, spared nerve injury; TGN, trigeminal neuron; TNF-α, tumor necrosis factor-α; TRP, transient receptor potential; VRT, ventral root transection. ** indicates supplementary information.

## Botulinum Neurotoxin: Pain Treatment

Botulinum toxin treatments have been demonstrated to be effective and are currently used for the treatment of chronic migraine, while clinical trials are ongoing for their use in other pain conditions (Burstein et al., 2014). The efficiency and potency of these treatments can be due to BoNT multiple actions in nociceptors, modulation of TRP channels, and synaptic transmission. BoNT/A has shown analgesic effects in both acute and chronic animal models of pain. However, such analgesic effects are still debatable, and this toxin cannot attenuate the spinal release of neuropeptides and the pain induced by injection of a high dose of capsaicin. This is probably due to the limited cleavage of SNAP-25 by BoNT/A, which fails to prevent the formation of a functional SNARE complex (Meng et al., 2009, 2014).

To improve the functional and analgesic effects of BoNTs, researchers have started to study additional BoNT serotypes and transgenic chimeras. In particular, BoNT/E cleaves 26 residues from SNAP-25 (compared with nine cleaved by BoNT/A) and may better prevent the formation of the SNARE complex (Meng et al., 2009; Wang et al., 2011). However, BoNT/E has a shorter half-life than BoNT/A (Wang et al., 2017). Therefore, researchers have generated a recombinant chimera of BoNT/A and BoNT/E which has shown analgesic effects for both mechanical and cold hypersensitivity in a spared nerve injury animal model of neuropathic pain, which is particularly refractory to current pain drugs. Indeed, a single injection of this chimera demonstrated long-lasting analgesic effects (up to 2 weeks) that were far superior to those of multiple injections of BoNT/A or pregabalin (Wang et al., 2017).

Despite these promising data, there are still challenges to be considered for the use of BoNTs in chronic pain. Some reports indicated the potential generation of antigens against BoNTs, which may explain the non-responsiveness to BoNTs in a handful of patients (Zuber et al., 1993; Stephan et al., 2014). Antigen generation may be reduced by developing novel BoNT formulations using alternative serotypes or different formulations (i.e., reducing protein load including adjuvants), or by minimizing exposure (i.e., increasing injection intervals and decreasing doses) (Bellows and Jankovic, 2019).

Several alternative serotypes are currently being explored, with BoNT/C appearing to be a promising substitute for BoNT/A, with similar efficacy and duration of action and, most importantly, demonstrated analgesic efficiency in BoNT/A-resistant patients (Eleopra et al., 1997, 2006). Similarly, BoNT/F showed positive results, although with shorter duration, in BoNT/A-resistant patients (Greene and Fahn, 1993). Different formulations are being tested, and Allergan Inc. developed a new BoNT/A formulation (Botox) containing less neurotoxin complex protein per unit, which was reported to generate fewer antigens in patients injected with the new formulation when compared with those injected with older ones (Jankovic et al., 2003).

BoNT treatment requires multiple cutaneous injections producing discomfort in patients that may require anesthesia (Kranz et al., 2006; Weiss and Lavin, 2009), as well as potential extravasation in blood vessels (Naumann and Jankovic, 2004; Roche et al., 2008) and undesired adverse effects such as hematoma, bruising, or muscular weakness, highlighting the importance of developing alternative delivery methods (Wyndaele and Van Dromme, 2002; Wollina and Konrad, 2005). Novel and diverse delivery methods are under robust investigation, including physical approaches such as transdermal and transepithelial delivery, chemical approaches for recruiting enhancers to increase permeability, or the use of liposomes, together with the previously mentioned method of creating recombinant forms for more precise delivery to target organs (Fonfria et al., 2018).

## Conclusion

Chronic pain is a debilitating condition with few treatment options. Since their identification in nociceptors, interest in the role of TRP channels in pain and as therapeutic targets has steadily increased. Small-molecule inhibitors directly targeting TRPV1 and TRPA1 have been tested in various clinical trials without success, due to adverse side effects. Here, we summarized how TRP channel expression and function can be indirectly modulated by BoNTs, which are regarded as effective and safe analgesics. In particular, BoNT/A has been demonstrated to change the expression and translocation of TRP channels in nociceptors, and its analgesic effects have been proven experimentally in different acute and chronic pain conditions, as well as in the clinical treatment of chronic migraine. We believe that the continuous improvement of BoNTs and a better understanding of their mechanisms of action, including those involving the regulation of TRP channels, will lead to the clinical treatment of additional chronic pain conditions.

## Author Contributions

C-KP and TB conceived and supervised the project. EJG, JJ, YHK, and C-KP wrote the manuscript. All authors contributed to the article and approved the submitted version.

## Conflict of Interest

The authors declare that the research was conducted in the absence of any commercial or financial relationships that could be construed as a potential conflict of interest.

## Publisher’s Note

All claims expressed in this article are solely those of the authors and do not necessarily represent those of their affiliated organizations, or those of the publisher, the editors and the reviewers. Any product that may be evaluated in this article, or claim that may be made by its manufacturer, is not guaranteed or endorsed by the publisher.
